# Coronin 1-dependent cell density sensing and regulation of the peripheral T cell population size

**DOI:** 10.1093/oxfimm/iqae002

**Published:** 2024-03-22

**Authors:** Tohnyui Ndinyanka Fabrice, Mayumi Mori, Jean Pieters

**Affiliations:** Biozentrum, University of Basel, Basel, Switzerland; Graduate School of Medicine, Kyoto University, Kyoto, Japan; Biozentrum, University of Basel, Basel, Switzerland

**Keywords:** coronin, T cells, cell population size regulation, T cell homeostasis, immunodeficiency, cAMP-Ca^2+^ signalling

## Abstract

The establishment and maintenance of peripheral T cells is important to ensure appropriate immunity. In mammals, T cells are produced in the thymus before seeding the periphery early in life, and thereafter progressive thymus involution impairs new T cell production. Yet, peripheral T cells are maintained lifelong at approximately similar cell numbers. The question thus arises: what are the mechanisms that enable the maintenance of the appropriate number of circulating T cells, ensuring that T cell numbers are neither too low nor too high? Here, we highlight recent research suggesting a key role for coronin 1, a member of the evolutionarily conserved family of coronin proteins, in both allowing T cells to reach as well as maintain their appropriate cell population size. This cell population size controlling pathway was found to be conserved in amoeba, mice and human. We propose that coronin 1 is an integral part of a cell-intrinsic pathway that couples cell density information with prosurvival signalling thereby regulating the appropriate number of peripheral T cells.

## Introduction

T cells are crucial for maintaining health, and do so by establishing and maintaining a pool of peripheral T cells that circulate through the body at near constant numbers, mostly in a so-called ‘naïve’ state, coming into action whenever danger appears in the form of infectious agents, foreign bodies or oncogenically transformed cells [1–4]. T cells originate from haematopoietic stem cells in the bone marrow and undergo selection in the thymus to ensure their ability to recognize foreign antigens while avoiding self-reactivity prior to export into the periphery. Once in the periphery, T cells can recognise foreign, non-self antigens through their cell surface-exposed T cell receptors (TCRs) that recognise these antigens in the form of peptides bound to Major Histocompatibility Complex (MHC) molecules (pMHC) presented by antigen presenting cells [5]. The total number of T cells in the periphery has been estimated to be in the range of 10^7^-10^8^ in mouse and 10^11^ in human [6, 7]. Both haematopoietic stem cell production, thymic selection as well as the establishment of peripheral T cells are relatively well understood processes [8, 9], and not the subject of this paper. Instead, we will address the question of how, throughout life and in the absence of new thymic output, appropriate numbers of T cells are being maintained.

Here, we argue that the population size of peripheral T cells is set and maintained through a cell population size-sensing mechanisms that is coordinated by coronin 1, a member of the evolutionarily conserved family of coronin proteins that is expressed in immune cells and to a far lesser degree in neurons [10, 11]. We review evidence suggesting a key role for coronin 1 in the regulation of peripheral T cell numbers through coupling T cell survival with a cell-intrinsic cell density-sensing mechanism [12, 13]. We further argue that such coronin-dependent T cell population size regulation is consistent with the ability to maintain a pool of clonally diverse T cells, a feature that is essential to maintain life-long immunity towards a diverse array of potential harmful entities, including pathogenic microbes and oncogenically transformed cells.

## Establishment and maintenance of the peripheral T cell population

T cells start their journey towards peripheral lymphoid organs as T cell progenitors originating from self-renewing stem cells residing in the bone marrow and transitioning into multipotent progenitors that become committed to the T cell lineages along their way to the thymus [14, 15, 8]. Upon entering the thymus, such T cell progenitors undergo different selection processes to ensure that those T cells that arrive in the periphery are both self-tolerant and useful for recognising foreign peptides in the context of molecules of the major histocompatibility complexes (MHC class I and II) [16, 17]. These selection processes include the survival of those thymocytes recognising host MHC molecules (positive selection), as well as the elimination of autoreactive thymocytes to avoid autoimmunity (negative selection) [18]. T cell selection in the thymus has evolved to be a stringent process aimed at avoiding potential harm imposed by inappropriately selected T cells; indeed, most thymocytes die via programmed cell death leaving only 2–5% of all T cell precursors to make it into the peripheral T cell population [18, 19]. Thymic selection also confers properties, including the ability to signal through the T cell receptor (TCR) and differential expression of genes implicated in the long-term survival of the T cells in the periphery [18, 20, 21].

**Figure 1.**
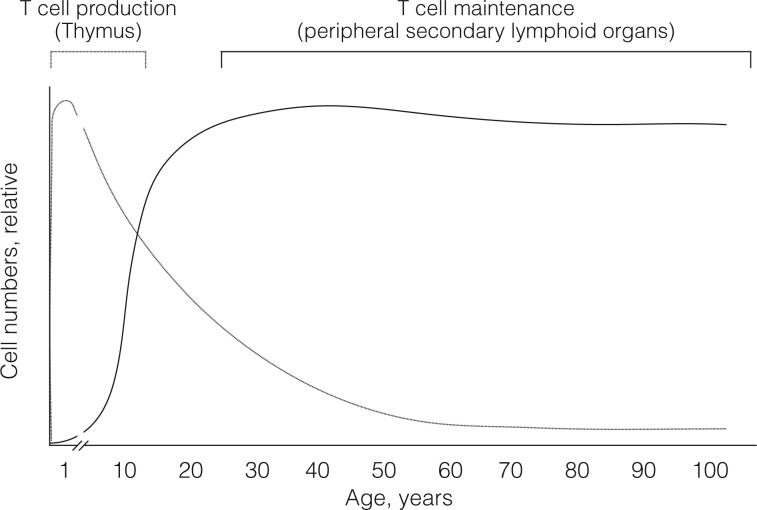
Thymic involution, T cell production and peripheral T cell maintenance. In humans, the thymus reaches its largest size within the first year of life, thereafter undergoing involution (dotted line, taken from [22, 23]), suggesting a short burst of T cell production early in life. Once produced and transferred into the periphery, T cells are being maintained long-term within peripheral secondary lymphoid organs (solid line), providing life-long immune protection, see also [6, 32, 74].

Within the scope of the here-discussed existence of a coronin-mediated cell-intrinsic mechanism regulating peripheral T cell numbers, the following should be noted: First, the thymus is most active in new-borns, and, in fact, starts to undergo involution after the first 12 months of life in humans and the first few post-natal weeks in mice ([22, 23], see also Fig. 1). Thymic involution appears to be conserved in all species that possess a thymus, suggesting that first, T cell production is most relevant early in life, and second, that thymic involution may provide evolutionary advantages [6, 22, 24–26]. Further, although thymic involution has been suggested to be involved in age-dependent immunosenescence [25], the immune system is active across an individual life’s history, and thymectomising adults does not affect lifespan [22, 27]; in fact, factors other than thymic involution have been proposed to underly age-associated immune dysfunction [28–31]. It could therefore well be that evolutionary pressure has ensured the generation of a broad T cell repertoire immediately after birth, which is also the timeframe when new-borns are being exposed to a myriad of different pathogens. Perhaps it is of advantage to coordinate peripheral T cell production within a narrow timeframe, thereby avoiding wasting resources later on in life, especially when new-borns can generate a T cell repertoire broad enough to suffice throughout the life span of the individual [22, 25].

**Figure 2.**
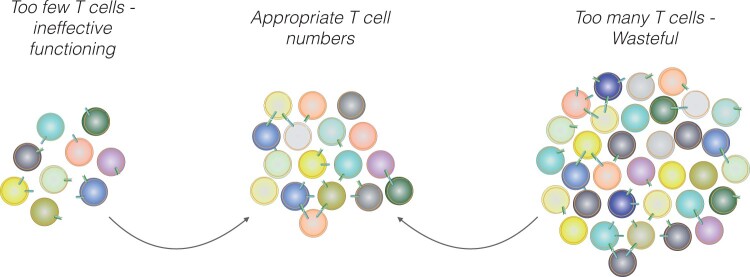
Maintenance of peripheral T cell numbers at appropriate cell densities. In case of too few T cells (left), the organisms are insufficiently protected against pathogenic challenges, while too many T cells (right) would result in unnecessarily wasting resources necessitating the existence of mechanisms regulating the appropriate T cell population size. Cell-cell interaction and signalling among T cells are depicted by receptor-ligand interactions.

This raises the second point we would like to address, namely the fact that peripherally circulating T cells are long-lived, with estimates ranging from several months in mice to decades in humans [1, 2, 32, 33], therefore suggesting the existence of a mechanism allowing not only the establishment but also the long-term maintenance of appropriate T cell numbers. Maintenance of appropriate T cell numbers is important, since T cells are likely to function optimally at defined cell densities: in case of too few circulating T cells and potential loss of TCR diversity, the individual may be at risk of insufficient protection from pathogens, while the presence of too many T cells may result in wasting of resources and/or harmful side effects (Fig. 2). Thus, with the peripheral T cell pool being generated early in life during a short burst of thymic activity, and the demise of the thymus starting soon after, there is a need for a mechanism that controls the appropriate numbers of T cells in the periphery to maintain the appropriate size and clonal diversity of the peripheral T cell population.

Here, we posit that signalling mechanisms coordinated by coronin 1, a member of the evolutionarily conserved family of coronin proteins, govern both the setting of the threshold density and maintenance of the peripheral T cell population at the appropriate numbers. Interestingly, this role for coronin 1 in establishing and maintaining the correct number of T cells within peripheral lymphoid organs was found to parallel the role for coronin expressed in lower eukaryotes in allowing the establishment of threshold cell densities, suggesting the existence of a coronin-dependent density-sensing mechanisms important for setting the threshold and maintenance of cell populations [13].

## Coronin 1 and the peripheral T cell pool size

**Figure 3.**
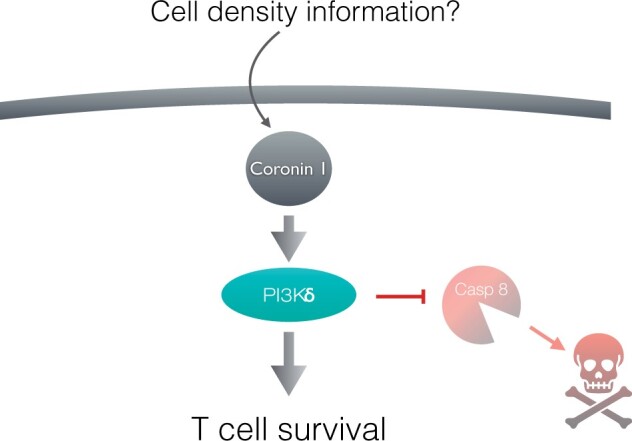
Peripheral T cell viability is maintained by coronin 1-dependent activation of PI3Kδ. Maintenance of PI3Kδ activity by coronin 1 prevents caspase 8 from activating cell death pathways. Whether or not coronin 1 acts downstream of a cell density-sensing pathway as drawn in this scheme is not known. Modified from Mori *et al*. [12].

**Figure 4.**
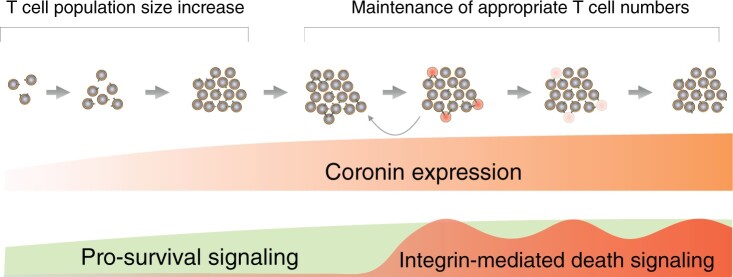
Establishment and maintenance of the appropriate peripheral T cell population size by coronin 1. At low densities (left), increased expression of coronin 1 provides prosurvival signalling thereby allowing the establishment of the appropriate T cell population size (middle). At the threshold cell densities, coronin 1 expression peaks, and any further population size increase results in cycles of cell death to return and maintain the cell population to its appropriate size (right). Modified from Ndinyanka Fabrice *et al*. [13]. Cell-cell interaction and signalling mediated by adhesion molecules cells are depicted by receptor-ligand interactions.

The notion that coronin 1 (also known as tryptophan aspartate containing coat protein, or P57 [10, 34]) functions as a regulator of peripheral T cell populations came from work analysing the consequences of ablation of the coronin 1-encoding gene in mice as well as the profound T cell-deficiency observed in individuals carrying mutations in the coronin 1-encoding gene [35–41]. Coronin 1, that is one of the most abundantly-expressed proteins in T cells [13], is a cytosolically expressed protein that partially associates with the plasma membrane in a cholesterol-dependent fashion [10, 42]. How, exactly, coronin 1 mediates T cell population size regulation is a matter of debate. Coronin 1 has been suggested to be involved in the regulation of F-actin dynamics [35, 39, 41]. However, F-actin–dependent leukocyte functions are not affected by deletion of coronin [43–45]. Alternatively, coronin 1 has been proposed to regulate T cell population sizes via Ca^2+^/cAMP-dependent pro-survival signalling [36, 38, 43–48]. More recent research suggest a key role for coronin 1 in coordinating the inhibition of cell death pathways with cell-intrinsic cell number-sensing to regulate the appropriate peripheral T cell population size as discussed below (see also Figs 3 and 4 and [12, 13]).

## Sustaining peripheral T cell survival by coronin 1-dependent inhibition of caspase 8 through maintenance of PI3Kδ activity

The peripheral T cell pool is established from recent thymic emigrants, that undergo further differentiation into mature naïve T cells in the periphery [49, 50]. The role of coronin 1 in establishing the appropriate numbers of peripheral T cells appears to be specific from the mature naïve T cell stage onwards, since coronin 1 is dispensable for the generation and/or survival of recent thymic emigrants [12, 48].

The finding that coronin 1 is dispensable for both thymic selection and egress also suggests that coronin 1-dependent peripheral T cell survival is distinct from the peptide MHC (pMHC):TCR and/or interleukin-7 (IL-7) signalling pathways [12] that are important for thymic selection and output [18, 51]. Indeed, the loss of peripheral T cells in coronin 1-deficient mice appears not to be due to any reduction in TCR- or IL-7-mediated proliferative capacity [12, 48]. Rather, coronin 1 maintains viability through promoting prosurvival and inhibiting cell death signalling, which is supported by data showing a requirement for coronin 1 in sustaining the viability of adoptively transferred naïve T cells into MHC class II-deficient mice, *i.e.* in mice lacking the stimuli acting through T cell receptors [12]. In fact, coronin 1 provides prosurvival signalling through maintaining phosphoinositide 3-kinase (PI3K) δ activity, thereby suppressing caspase 8-mediated apoptosis ([12] and Fig. 3).

The involvement of coronin 1 in PI3Kδ activation is specific for mature naïve T cells and not observed in thymocytes or recent thymic emigrants [12, 48], although the molecular players involved in the activation of the coronin 1/PI3Kδ pathway upon maturation from recent thymic emigrants remain unclear. Also, although cell number-sensing feedback may serve as a direct or indirect regulator of coronin 1-dependent maintenance of PI3Kδ activity, the underlying molecular mechanisms remain currently unclear. In this light, it is interesting to note that PI3K activity is known to be regulated by Gβγ subunits of trimeric G proteins [52, 53]. Hence, given the homology of coronin proteins to the beta subunit of trimeric G proteins and their involvement in G protein signalling [43, 47, 54–57], future work may well consider to analyse a potential role for coronin 1 in PI3Kδ activation through acting as a Gβγ mimic, thereby maintaining constitutive levels of activated PI3Kδ. Such a mechanism would also be consistent with the above-mentioned possibility of coronin 1 acting to rescue T cells from default death in a cell-intrinsic manner.

## Coronin 1-dependent cell-intrinsic T cell survival and the tuning of the peripheral T cell population size

Suppression of cell death alone, as described above, is insufficient to explain how the threshold population size is set, below which T cells should expand and above which the population should initiate contraction. The question then is: how do T cells perceive and tune their population size to achieve population robustness? This question is also relevant given the short burst of thymic activity relative to the lifespan of the individual [22, 23], as well as the ‘sizing’ of individuals from young age to adult life, and suggests the need for a mechanism allowing the sensing as well as the maintenance of the appropriate T cell population size.

For many cell types, viability has been suggested to depend on continued signals from other cells to avoid death [58]. Here, we postulate that the prosurvival activity of coronin 1 may be an integral part of a hitherto uncharacterised cell-intrinsic survival and cell density information-processing pathway that is key to providing a cell-intrinsic death rescue signal within circulating peripheral T cells. In this scenario, the ***absence*** of signalling (i.e. until threshold densities are reached), rather than the ***presence*** of signals derived from other cells, would allow coronin 1 to promote cell survival. Of note, coronin 1 expression increases with cell density (becoming one of the most abundant proteins at threshold densities [13]). Coronin 1-dependent cell-intrinsic prosurvival signalling is counterbalanced at cell densities above threshold, when coronin 1 expression becomes limiting, resulting in cell death being executed through kin-to-kin signalling to maintain the appropriate cell population size (see below and Fig. 4).

Thus, apart from keeping naïve T cells alive through inhibition of caspase 8 through PI3Kδ activation, coronin 1 plays a central role in setting and maintaining the T cell population size by regulating cell density-dependent prosurvival signalling until the appropriate cell population size is reached (Fig. 4). Coronin 1 does so through intracellular retention of the T cell adhesins lymphocyte function-associated antigen 1 (LFA-1) and intercellular adhesion molecule 1 (ICAM1). For reasons not yet understood, when coronin 1 concentrations in the cell become limiting (at threshold densities), LFA-1/ICAM1 cannot anymore be retained intracellularly thus appearing at the cell surface where their ligation through kin-to-kin interactions results in initiation of apoptosis [13]. In this scenario, the (maximal) cellular concentration of coronin 1 at T cell threshold density would set the surface expression levels of LFA-1/ICAM1, and upon further T cell expansion, the resulting increased frequencies of cell-to-cell contact will lead to a higher rate of adhesin-mediated cell death signalling thus maintaining the cell population at its appropriate density and size ([13] and Fig. 4).

## Molecular mechanism regulating coronin 1-dependent T cell population size regulation

What may be the molecular function via which coronin 1 coordinates the above-described cell-intrinsic survival and T cell population sizes? We propose that coronin 1 does so through regulation of cAMP/Ca^2^^+^ signalling, a function that would also be consistent with the observed cell-density dependent modulation of Ca^2+^ signalling by coronin 1 [13]. Furthermore, modulation of cAMP/Ca^2+^ signalling by coronin 1 has been reported in multiple cell types, including T cells and neurons [36, 38, 43–46, 59].

A role for coronin 1 in regulating the cAMP pathway would also explain the striking phenotype of coronin 1-deficient mice being resistant towards auto- and alloimmune challenges, given the known role for cAMP in promoting immune suppression [36, 47, 60–64]. Furthermore, a function for coronin 1 in the regulation of cAMP signalling would be consistent with data showing that also in amoeba, the coronin 1 homologue, coronin A, regulates cAMP signalling [57]. How, exactly, coronin 1 may regulate cAMP/Ca^2+^ signalling remains unclear, and may be related to its structural homology to components of the trimeric G protein pathway involved in G protein-coupled receptor-mediated signalling [54–56, 65, 66].

## Coronin 1-dependent T cell population size control and T cell diversity

For the peripheral T cell population to function adequately, population robustness is achieved only when the appropriate T cell population size is matched by robust clonal diversity. While coronin 1 appears to be dispensable for the processes that shape the T cell repertoire during thymic selection, its role in kin-to-kin population-intrinsic size regulation in the periphery could be crucial for maintaining robust T cell clonal diversity [12, 48, 67, 68]. Interestingly, recent work, based on mathematical modelling and analysis of experimental datasets, suggests that once a diverse pool of T cells is established early in life, the peripherally circulating mature naïve T cells regulate their pool size in a cell-autonomou*s* manner [32], a notion that would be consistent with the cell-intrinsic role for coronin 1 in peripheral T cell survival. Further, cell-intrinsic (here akin to population-intrinsic) signals, where T cells are capable of kin-to-kin (T cell-to-T cell) communication, retains the simplicity and scalability required for the sensing and adjustment of population density and diversity to achieve robustness.

## An evolutionarily-conserved coronin-dependent cell density sensing pathway

Coronin-encoding genes are widely expressed in eukaryotes, with the possible exception of plants [40, 69]. In light of the above-described role for coronin 1 in the regulation of peripheral T cell populations, it is interesting to note that the coronin 1 homologue in the slime mould *Dictyostelium discoideum*, coronin A [40, 55], appears to perform a similar role in sensing and setting cell population density in a process that appears to depend on cell-to-cell adhesins, as is the case for peripheral T cell population size regulation [13]. Dictyostelia are early eukaryotes that switch between uni- and multicellular life forms [70, 71]. Whether or not coronin genes have evolved to regulate multicellularity in diverse eukaryotes will be an interesting question to address in future research.

## Coronin 1-dependent peripheral T cell population size regulation: open questions

The question of ‘niche’ and ‘space’ for the peripheral T cell pool has been raised before [50, 72, 73] although a molecular understanding of the concepts of space and niche has been lacking. Here, we propose coronin 1 activity to be a basis for regulating the T cell ‘niche’ and ‘space’ through its function in setting the threshold and maintaining appropriate T cell population sizes as well as T cell clonal diversity. In future research, the following open questions may be addressed to further define the role of coronin 1 in T cell population size regulation: What is the exact relationship between coronin 1 expression and cell population size? Would cells require increased coronin 1 expression to expand to higher numbers, or, alternatively, do higher cell numbers induce higher expression of coronin 1? Moreover, what are the mechanisms via which T cells perceive the need to regulate the expression of coronin 1 to achieve steady-state homeostatic population size? Could such sensing mechanism be similar to or distinct from T cell population expansion and contraction that characterizes T cell mediated immune responses? Is deregulation of the coronin pathway involved in leukemic transformation?

Together, addressing these questions may allow a detailed molecular understanding of how T cells, that have been suggested to live forever [33], may be sustained long-term in a manner that allows the continued control of pathogenic challenges. Further, given the widespread expression of coronin-encoding genes in eukaryotes, research on the mechanism of action of members of the coronin proteins may perhaps be relevant for a better understanding of the molecular mechanisms underlying eukaryotic multicellularity.

## Data Availability

No data available.
